# Deleterious *GRM1* Mutations in Schizophrenia

**DOI:** 10.1371/journal.pone.0032849

**Published:** 2012-03-20

**Authors:** Mohammed Akli Ayoub, Dora Angelicheva, David Vile, David Chandler, Bharti Morar, Juleen A. Cavanaugh, Peter M. Visscher, Assen Jablensky, Kevin D. G. Pfleger, Luba Kalaydjieva

**Affiliations:** 1 Western Australian Institute for Medical Research/UWA Centre for Medical Research, University of Western Australia, Perth, Australia; 2 Centre for Clinical Research in Neuropsychiatry, The University of Western Australia, Perth, Australia; 3 Research School of Biological Sciences, Australian National University, Canberra, Australia; 4 Queensland Institute for Medical Research, Royal Brisbane Hospital, Brisbane, Australia; 5 School of Psychiatry and Clinical Neurosciences, The University of Western Australia, Perth, Australia; University of Illinois-Chicago, United States of America

## Abstract

We analysed a phenotypically well-characterised sample of 450 schziophrenia patients and 605 controls for rare non-synonymous single nucleotide polymorphisms (nsSNPs) in the *GRM1* gene, their functional effects and family segregation. *GRM1* encodes the metabotropic glutamate receptor 1 (mGluR1), whose documented role as a modulator of neuronal signalling and synaptic plasticity makes it a plausible schizophrenia candidate. In a recent study, this gene was shown to harbour a cluster of deleterious nsSNPs within a functionally important domain of the receptor, in patients with schizophrenia and bipolar disorder. Our Sanger sequencing of the *GRM1* coding regions detected equal numbers of nsSNPs in cases and controls, however the two groups differed in terms of the potential effects of the variants on receptor function: 6/6 case-specific and only 1/6 control-specific nsSNPs were predicted to be deleterious. Our in-vitro experimental follow-up of the case-specific mutants showed that 4/6 led to significantly reduced inositol phosphate production, indicating impaired function of the major mGluR1signalling pathway; 1/6 had reduced cell membrane expression; inconclusive results were obtained in 1/6. Family segregation analysis indicated that these deleterious nsSNPs were inherited. Interestingly, four of the families were affected by multiple neuropsychiatric conditions, not limited to schizophrenia, and the mutations were detected in relatives with schizophrenia, depression and anxiety, drug and alcohol dependence, and epilepsy. Our findings suggest a possible mGluR1 contribution to diverse psychiatric conditions, supporting the modulatory role of the receptor in such conditions as proposed previously on the basis of in vitro experiments and animal studies.

## Introduction

Metabotropic glutamate receptor 1 (mGluR1), the protein product of the *GRM1* gene, is a Family C G protein-coupled receptor (GPCR) belonging to Group I of mGluRs, along with mGluR5. In addition to the seven transmembrane helical structure, conserved throughout all GPCRs, Class C receptors are also characterised by a large bi-lobed ligand binding domain linked to the transmembrane region by a cysteine-rich domain, as well as a large intracellular C-terminal tail [1]. mGluR1 is predominantly expressed post-synaptically in neurons of the hippocampus, hypothalamus, thalamus, amygdala, basal ganglia, cerebellum and spinal cord [2]–[5], where it modulates N-methyl-D-aspartate (NMDA), α-amino-3-hydroxy-5-methyl-4-isoxazolepropionic acid (AMPA) and γ-aminobutyric acid (GABA) receptor activity [6].

Pharmacological activation of mGluR1, leading to activation of phospholipase C and consequent phosphoinositide hydrolysis, has been shown to facilitate NMDA receptor responses in cortical neurons and the CA3 area of the hippocampus [6]–[9]. The receptor also plays a critical role in synaptic plasticity, memory and learning [10]–[13] by regulating local dendritic protein synthesis in functional interaction with the fragile X mental retardation protein (FMRP) [14]–[18]. Hypofunction/dysregulation of glutamatergic signalling is one of the dominant concepts of schizophrenia pathogenesis, originally based on observations that NMDA receptor antagonists can induce psychotic symptoms and cognitive deficits closely resembling those in schizophrenia, and later supported by findings of altered NMDA receptor subunit composition and changes in NMDA receptor-related postsynaptic proteins in schizophrenia brains [19]–[23]. While the experimentally documented mGluR1 functions could be related to the dysfunctional glutamatergic signalling and impaired synaptic plasticity underlying the cognitive deficit of affected individuals [19]–[23], *GRM1* has not emerged as a schizophrenia candidate gene from linkage and association studies. Until recently, its possible involvement was suggested only by observations of altered expression levels in post-mortem schizophrenia brains [24], [25], where increased mGluR1 expression in the prefrontal cortex has been interpreted as a compensatory change to the NMDA receptor hypofunction [24], as well as by the deficits in sensorimotor gating (prepulse inhibition of acoustic startle) in *Grm1* knock-out mice [26] similar to those seen in schizophrenia patients.

In a recent study, Frank et al. [27] examined common variants in multiple genes encoding post-synaptic density proteins, in parallel with a search for rare mutations specifically targeting “hub” genes involved in glutamate neurotransmission. Sequencing analysis in 503 schizophrenia and 263 bipolar cases, and 538 controls identified an unusual clustering of rare non-synonymous variants in *GRM1*, in the small gene region encoding the functionally important cysteine-rich domain, which mediates receptor dimerization and activation upon ligand binding [27]. This was the first study to implicate directly rare *GRM1* mutations in the pathogenesis of psychotic illness.

Here, we examined the occurrence, inheritance and functional effects of rare *GRM1* nsSNPs in a clinically and neurocognitively characterised sample of 450 schizophrenia cases and 605 controls.

## Materials and Methods

### Ethics Statement

All subjects had provided written informed consent and the study was approved by the Human Research Ethics Committee of The University of Western Australia. The capacity of the patients to provide informed consent was established through a structured interview, and was also confirmed by their treating psychiatrists.

### Subjects

The cases, 348 male and 102 female patients of European descent (∼75% Anglo-Irish), were recruited from consecutive admissions to psychiatric hospitals or community mental health centres as part of the West Australian Family Study of Schizophrenia (WAFSS). Diagnostic assessment was based on standardized interviews using the Schedules for Clinical Assessment in Neuropsychiatry [28] and scored with the OPCRIT algorithm [29]. Videorecorded interviews and clinical charts were independently reviewed by two senior clinicians who assigned consensus research ICD-10 and DSM-IV diagnoses. The 605 healthy controls included 265 WAFSS participants, recruited by random sampling from local telephone directories or among Red Cross blood donors; the remainder were samples from the “Aussie Normals” collection (http://www.neura.edu.au/content/aussie-normals).

All WAFSS participants completed a battery of neurocognitive tests as described [30]. In brief, these included pre-morbid IQ (National Adult Reading Test, NART); current IQ (Shipley Institute of Living Scale, SILS); verbal memory (Rey's Adult Verbal Learning Test immediate and delayed word recall, RAVLT-IW and RAVLT-DW); and sustained attention (Continuous Performance Task identical pairs and degraded stimulus, CPTip and CPTds). Performance data from the multiple neurocognitive domains were integrated into a cognitive deficit (CD) and cognitively spared (CS) composite continuous trait, using a grade of membership (GoM) model [30]–[32]. Based on a statistically defined cut-off in the scores, patients were assigned to a CD or CS cluster, and a small residual non-CD/non-CS group.

### Re-sequencing of the *GRM1* gene

The eight coding exons and at least 100 bp of flanking intronic sequences of the longest *GRM1* transcript (NM_000838), a total of 5780 bp (3585 bp of which was coding), were PCR-amplified from genomic DNA extracted from fresh blood using ten amplicons. Primers (Table S1) were designed using the Primer3 software (http://frodo.wi.mit.edu/primer3/). PCR reactions and Sanger sequencing of both strands were performed at the Australian Genome Research Facility (Brisbane, Queensland) in a 384-well plate format following standard procedures. The sequencing data were analysed with the Sequencher 4.8 software, followed by visual examination of the identified variants in the chromatograms. Technical replication of all identified novel changes in the coding sequence was done by Sanger sequencing of both DNA strands in newly aliquoted DNA samples.

### Bioinformatics analysis of detected sequence variants

The potential effect of non-synonymous sequence variants on protein structure and function was evaluated using four different prediction programs: PolyPhen2 [33] (http://genetics.bwh.harvard.edu/pph2/); SIFT [34] (http://sift.jcvi.org/); Pmut [35] (http://mmb.pcb.ub.es/PMut/); and PhD-SNP [36] (http://gpcr2.biocomp.unibo.it/cgi/predictors/PhD-SNP/PhD-SNP.cgi). Intronic and coding synonymous variants were assessed for potential effects on splicing using Human Splicing Finder [37] (http://www.umd.be/HSF/) and ESEFinder [38] (http://rulai.cshl.edu/tools/ESE/).

### Family studies

The families of patients carrying nsSNPs were approached through the proband to obtain informed consent for additional studies. During a home visit by members of the research team, family members completed diagnostic interviews and neurocognitive tests. Data on family history of neuropsychiatric disorders were obtained through interviews with key informants. The nsSNP segregation pattern was analysed by sequencing the respective exons in the available DNA samples of first degree relatives.

### Plasmid construction and reagents for *in vitro* analyses of mGluR1 function

Wild-type (wt) *GRM1* cDNA synthesis, mutagenesis introducing the variants of interest, N-terminal tagging with a Flag tag, and subcloning into the pcDNA3.1 vector was performed by GENEART (www.geneart.com; Regensburg, Germany). The plasmid coding for the glutamate transporter EAAC1 (gene symbol *SLC1A1*) was generously provided by Dr. Jean-Philippe Pin (IGF Montpellier, France). Quisqualic acid (Quisqualate) was from Tocris Bioscience (Ellisville, Missouri, USA). The purified Mouse Anti-mGluR1 antibody was purchased from BD Transduction Laboratories (Franklin Lakes, NJ, USA).

### Cell culture and transfection

COS-7 cells were maintained at 37°C, 5% CO_2_ in complete medium (Dulbecco's modified Eagle's medium (DMEM) containing 0.3 mg/ml glutamine, 100 IU/ml penicillin, and 100 µg/ml streptomycin) supplemented with 10% fetal calf serum (FCS) (GIBCO BRL, Carlsbad, CA). Transient transfections were carried out in a 96-well plate using GeneJuice (Merck, Kilsyth, Australia). Briefly, for each well, 150 ng of mGluR1-coding plasmid (wild-type and mutants) and 100 ng of EAAC1-coding plasmid were incubated for 20 minutes at room temperature with a mix of 0.5 µl of GeneJuice and 49.5 µl of serum-free DMEM (pre-incubated at room temperature for 5 minutes). Cells (10^5^ in 150 µl/well) in DMEM supplemented with 10% FCS were then incubated with the final DNA-GeneJuice mix (50 µl/well). 24 h post-transfection, the medium was replaced by DMEM-Glutamax (GIBCO BRL, Carlsbad, CA).

### Inositol monophosphate (IP_1_) production measurements

IP_1_ accumulation was measured in white 96-well plates (10^5^ cells/well) using the IP-One Tb kit (Cisbio Bioassays, Bagnol sur Ceze, France). The cells were incubated for 45 minutes at 37°C in the stimulation buffer (10 mM HEPES, pH 7.4, 1 mM CaCl_2_, 0.5 mM MgCl_2_, 4 mM KCl, 146 mM NaCl, 5.5 mM glucose, and 50 mM LiCl) with or without 10 µM of the glutamate agonist Quisqualate used to activate the receptor. The cells were then lysed by adding the HTRF® assay reagents, the Terbium Cryptate-labeled anti-IP_1_ antibody, and the d2-labeled IP_1_ analog (diluted in the conjugated lysis buffer containing 1% Triton X-100). The assay was incubated for 1 hour at room temperature. After excitation at 340 nm, Terbium Cryptate fluorescence and the time-resolved FRET signal were measured at 620 and 665 nm respectively using an EnVision 2102 multilabel plate reader (PerkinElmer, Glen Waverley, Victoria, Australia).

### Enzyme-linked immunosorbent assay (ELISA)

COS-7 cells were washed once with phosphate-buffered saline (PBS), fixed for 10 minutes at room temperature with 4% paraformaldehyde and blocked with PBS containing 1% FCS. The receptor labelling was performed by incubation for 30 minutes at room temperature with 250 ng/ml (dilution 1∶1000) of purified mouse Anti-mGluR1 antibody in PBS containing 1% FCS. The cells were washed three times and incubated for 30 minutes with 0.5 µg/ml of horseradish peroxidase-conjugated anti-mouse secondary antibody (Amersham Pharmacia, NJ, USA) in PBS containing 1% FCS and washed three times again. For the detection of bound antibody, we used 10 µl/well of SuperSignal substrate (Pierce, Rockford, IL, USA) in 90 µl of PBS. Luminescence was measured in a VictorLight plate reader (PerkinElmer).

### Statistical analysis of functional data

Individual Quisqualate-induced IP_1_ responses and ELISA signals were expressed as percentages of the corresponding values obtained with wt mGluR1 in the same experiment. Mean percentages from four independent experiments carried out in triplicate were used for statistical analysis using a one sample t test comparing to the expected value of 100% (Prism 5 software, GraphPad, La Jolla, CA, USA).

## Results

High quality sequencing data were obtained for 1032 of the 1055 samples (443 cases and 589 controls) with minor variation in the numbers for the different *GRM1* exons (from 437 to 447 for cases and from 578 to 594 for controls).

### General characteristics of the detected variation

We identified a total of 47 variants, all of which were nucleotide substitutions, mostly (32/47) occurring in a single individual in the heterozygous state. 41 were coding SNPs and, of these, 16 were non-synonymous variants (Table S2).

**Figure 1:**
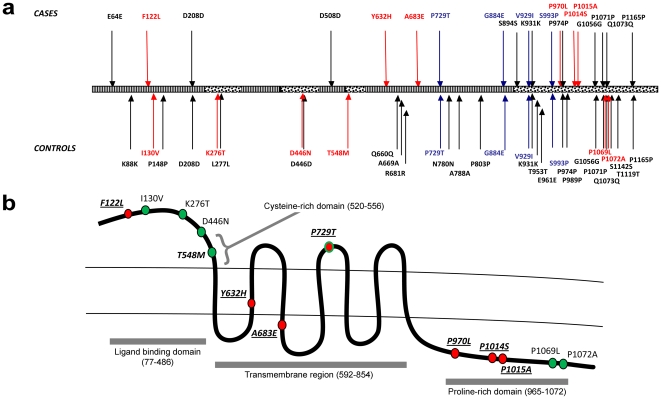
Distribution of the coding mutations in the *GRM1* gene and the encoded mGluR1 protein. (**a**) All coding mutations relative to gene structure, with *GRM1* exons 1 to 8 shown in alternating shaded bars. Top panel: changes found in patients; bottom panel: changes found in controls. Colour coding: red - rare missense variants; blue - common missense variants (MAF>1%), black- synonymous variants. (**b**) Non-synonymous coding changes relative to the mGluR1 receptor protein domains (shown as grey bars). Colour coding: red circles – case-specific, green circles – control specific, red circles with green outline – detected in cases as well as controls. Mutation predicted by bioinformatics programs to have a deleterious effect on protein function are italicised and underlined.

The detected changes were spread across the *GRM1* gene, with a significant proportion in the 3′ end (Table S2, Figure 1a). The overall diversity in the coding sequence was largely accounted for by the variable exon 8, which harboured 46% (19/41) of all coding and 44% (7/16) of the non-synonymous mutations, including the five known highly polymorphic coding SNPs: S993P, K931K, G1056G, P1071P and P1165P. *GRM1* exon 8 encodes most of the cytoplasmic C terminal tail of mGluR1, which mediates its interactions with multiple intracellular protein partners [39].

### Potential deleterious effects of the detected variants

Neither the intronic nor any of the synonymous coding mutations were predicted by Human Splicing Finder and ESEfinder to result in aberrant splicing.

Half of the nsSNPs (8/16), both rare and common, were classified as benign by all bioinformatics programs used for the prediction of potential functional effects (Table 1). The predictions for the remaining variants differed significantly between programs (Table 1), therefore further consideration was given to those classified as deleterious either with a high probability score by any program, or by more than one program regardless of the score. The best agreement was seen for the A683E mutation, carried by a single individual, and for the known SNP rs41305288 (P729T), present in the heterozygous state in 17 subjects - both were predicted to be deleterious by 3 of the 4 programs.

**Table 1:** Non-synonymous mutations and their predicted effects on mGluR1 function.

Mutation	Protein domain#	Bioinformatics prediction programs			
		PolyPhen-2	SIFT	PMut	PhD-SNP
**Cases only**					
F122L	LBD	**probably 0.950**	tolerated 0.15	neutral-5	**disease 6**
Y632H	TM2	**probably 0.999**	tolerated 1.00	neutral-5	disease 2
A683E	ICL2	**probably 0.998**	tolerated 0.10	**pathological-8**	**disease 6**
P970L	PRD	benign 0.021	**deleterious 0.03**	pathological-2	neutral 7
P1014S	PRD	benign 0.001	**deleterious 0.03**	neutral-5	neutral 8
P1015A	PRD	**probably 0.986**	tolerated 0.10	neutral-3	neutral 8
**Controls only**					
I130V	LBD	benign 0.004	tolerated 0.56	neutral-9	neutral 7
K276T	LBD	benign 0.180	tolerated 0.09	neutral-3	neutral 6
D446N	LBD	possibly 0.463	tolerated 0.46	neutral-4	neutral 8
T548M	CRD	**probably 0.996**	tolerated 0.14	neutral-4	neutral 8
P1069L*	PRD	benign 0.001	tolerated 0.51	pathological-1	neutral 6
P1072A	PRD	benign 0.019	tolerated 0.81	neutral-6	neutral 7
**Cases & controls**					
P729T*	ECL2	**probably 0.999**	**deleterious 0.01**	neutral-8	**disease 6**
G884E*	Cter	benign 0.023	tolerated 0.42	pathological-4	neutral 2
V929I*	Cter	benign 0.001	tolerated 0.00	neutral-9	neutral 6
S993P*	PRD	benign 0.009	tolerated 0.08	neutral-7	neutral 9

Prediction programs' scoring: PolyPhen-2 Bayes posterior probability that mutation is deleterious;

SIFT scores <0.05 indicate deleterious effect; PMut and PhD-SNP reliability of predictions 1-low, 9-high.

* known SNPs.

# Protein domains: LBD, ligand-binding domain; CRD, cysteine-rich domain; TM, transmembrane domain; ICL, intracellular loop; ECL, extracellular loop; Cter, C terminal tail; PRD, proline-rich domain in C terminal tail.

### *GRM1* sequence variation relative to affection status

We identified nine case-specific coding variants (synonymous and non-synonymous, Table S2), i.e. 2% of schizophrenia patients carried one. None are listed in dbSNP build 134, which incorporates data on rare mutations detected in the “1000 genomes” project. The number of control-specific coding variants was 21 (13 also listed in dbSNP 134), i.e. 3.6% of controls had one (p = 0.19).

The nsSNPs were also equally distributed between cases and controls (Figure 1b), with six detected in each group (1.3% of cases and 1% of controls, p = 0.57). SNP rs41305288 (P729T) was present in the heterozygous form in 6 cases (1.35%) and 11 controls (1.87%) (p = 0.46).

The only marginally significant difference apparent from these analyses concerned the distribution of rare potentially deleterious nsSNPs (Table 1, Figure 1b). All six in the schizophrenia group and only one in the controls were predicted to affect receptor function, i.e. 1.35% of affected subjects and 0.17% of controls carried a potentially harmful mutation (p = 0.047). The case-specific deleterious nsSNPs occurred in different parts of the gene, with one resulting amino acid substitution in the mGluR1 ligand- binding domain, two in the transmembrane region (including intracellular loop 2) and three in the proline-rich domain of the intracellular C-terminal tail.

### Phenotypes and family segregation patterns

**Figure 2:**
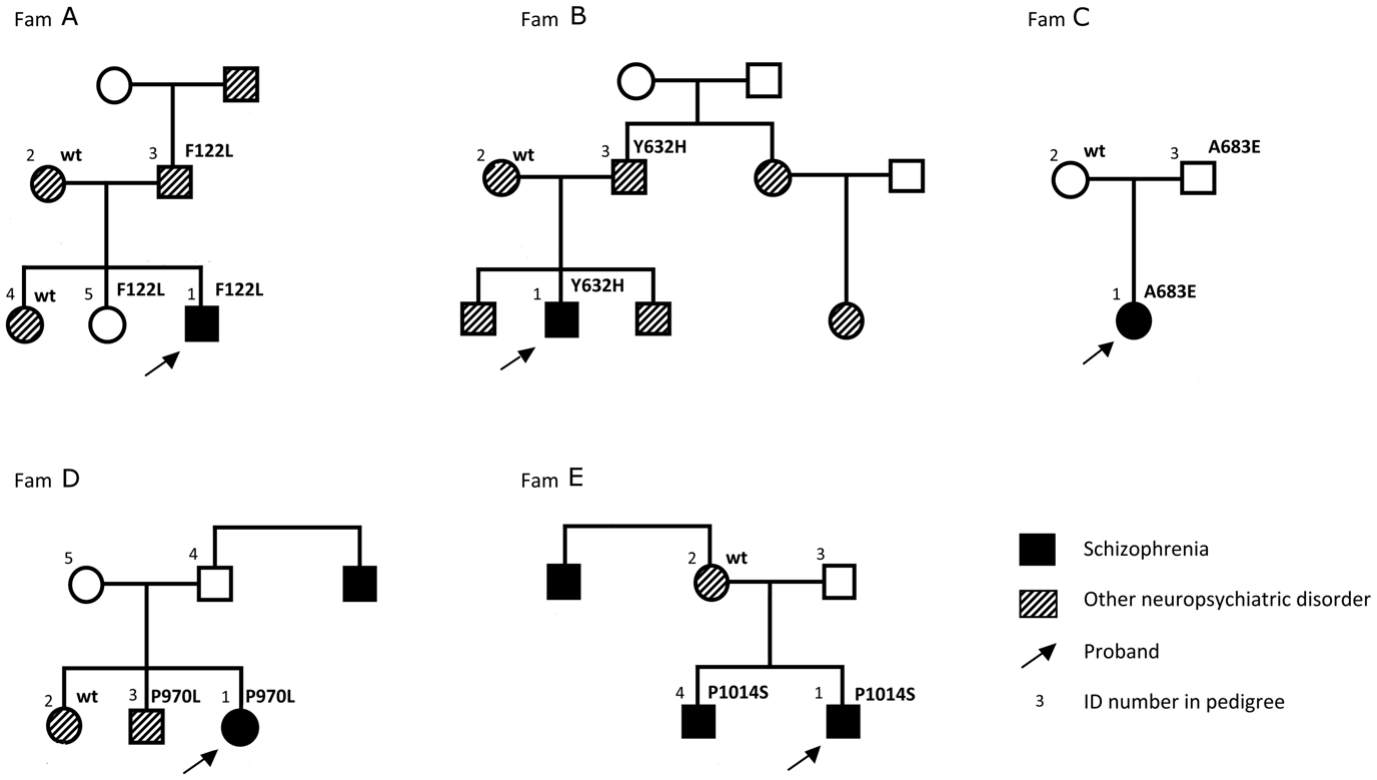
Structure, neuropsychiatric morbidity and mutation segregation in families of schizophrenia probands with deleterious *GRM1* variants.

The affected subjects carrying potentially deleterious nsSNPs in *GRM1* were distributed between diagnostic categories and neurocognitive clusters (Figure 2). Four had been classified as schizophrenia (mutations F122L, A683E, P1014S and P1015A), and two as schizoaffective disorder (mutations Y632H and P970L). The schizophrenia patients, carrying mutations A683E and P1014S, had been assigned to the cognitive deficit (CD) cluster, the remaining cases belonged to the cognitively spared cluster.

Additional information on family history and current status of 1^st^-degree relatives was collected, and genotyping performed for five potentially deleterious nsSNPs (all except P1015A). In addition to the probands with clinically diagnosed schizophrenia, 4 of these 5 families had multiple cases of other neuropsychiatric disorders, such as epilepsy, Asperger syndrome, anxiety, depression and substance abuse (Figure 2).

None of the five nsSNPs genotyped in family members was a *de novo* mutation, all were inherited. The family segregation analysis (Figure 2) showed that, in addition to the schizophrenia probands, *GRM1* mutations (e.g. F122L, Y632H and P970L) were also present in 1^st^ degree relatives with other neuropsychiatric conditions not limited to psychosis, suggesting a pleiotropic effect. F122L, A683E were also found in relatives completely free of psychiatric morbidity and with normal cognitive function, pointing to incomplete penetrance.

### Functional data

The major mGluR1-mediated G protein signalling pathway, resulting in production of inositol phosphates, was examined in COS-7 cells transiently expressing wild-type or mutant mGluR1. The utilized assay measured the production of inositol phosphate (IP_1_) in response to treatment with the glutamate agonist Quisqualate. To avoid saturation and desensitization of the receptor by endogenously produced glutamate, the cells were co-transfected with the EAAC1 transporter, the *in vivo* neuronal function of which is to terminate the post-synaptic action of glutamate by removing it from the synaptic cleft. We compared IP_1_ production in the cells expressing wild-type mGluR1 to that in cells expressing the case-specific non-synonymous changes and the known P729T polymorphism. The control-specific T548M was not included in these analyses as it was previously shown in a similar experimental design to have no functional consequences [27].

**Figure 3:**
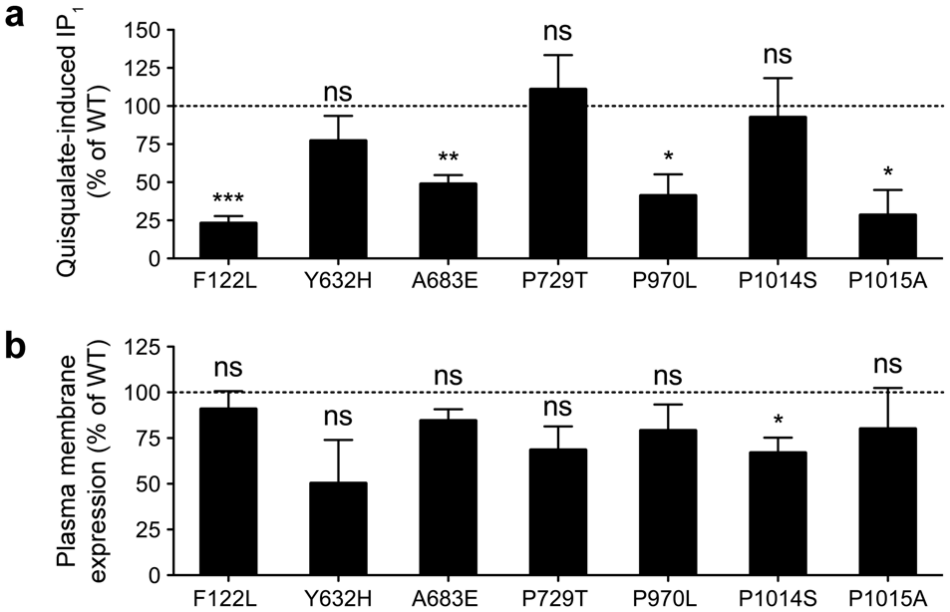
Functional analysis and relative plasma membrane expression of mGluR1 mutants in COS-7 cells. Cells transiently expressing mGluR1, wild-type (WT) or possessing the different mutations as indicated, were assayed for Quisqualate-induced inositol phosphate production using the IP-One assay (**a**) or plasma membrane expression using ELISA with anti-mGluR1 antibody on intact cells (**b**). With the IP-One assay, cells were stimulated with Quisqualate (10 µM) for 45 minutes and data were normalized to percentage of Quisqualate-induced IP_1_ production in cells expressing mGluR1-WT (**a**). Similarly, the ELISA signals were normalized to percentage of plasma membrane expression of the mGluR1-WT (**b**). Data are Mean ± SEM of four independent experiments carried out in triplicate. *** P<0.001, ** P<0.01, * P<0.05, ns P>0.05.

These analyses revealed a dramatic reduction in signalling efficacy caused by four of the mGluR1 mutants: F122L, A683E, P970L and P1015A compared to the wild-type receptor (Figure 3a). In contrast, no significant effect on inositol phosphate signalling efficacy was observed with the Y632H and P1014S mutations, or the P729T polymorphism in this *in vitro* cell system. Note that we did not see any specific cAMP signalling response resulting from mGluR1 activation by Quisqualate from any of the receptors, including wild-type (data not shown).

Since the observed signalling differences could be due to differing plasma membrane expression, we compared levels of the mutant receptors at the cell surface relative to wild-type. We performed an ELISA assay, using an anti-mGluR1 antibody, in parallel to the functional IP_1_ assay. We found that none of the mutations that affected signalling efficacy (F122L, A683E, P970L or P1015A) significantly affected plasma membrane expression. Thus, the observed decrease in Quisqualate-induced IP_1_ production with these mutations can be attributed to a reduction in mGluR1 signalling function in these cells. The P1014S mutation that did not affect signalling efficacy did however result in a significant reduction in cell surface expression by 33% (Figure 3). The Y632H mutation reduced cell surface expression by 50%, but this was not statistically significant due to high experimental variability (n = 4). The P729T polymorphism did not significantly affect either signalling efficacy or cell surface expression.

## Discussion

The modest success of genome-wide association studies, the disputed issue of “missing heritability”, and the advent of increasingly affordable large-scale sequencing technologies have shifted the paradigm in genetic studies of mental illness from the “common disease – common variants” concept to multiple rare mutations in diverse genes, or a combination of the two, ultimately converging to a manageable number of pathways of molecular pathogenesis. Two recent studies [40], [41] carried out exome sequencing in small numbers of sporadic schizophrenia cases and found a predominance of *de novo* mutations in multiple genes, most of which have not been implicated in schizophrenia previously, while others targeted candidate genes encoding synaptic proteins [42] or “hub” genes involved in glutamate signaling [27]. In this study, we performed Sanger sequencing of a single gene, *GRM1*, in a sample of well-characterized schizophrenia cases and controls from the West Australian Family Study of Schizophrenia. While the detected rare nsSNPs showed no difference between cases and controls in terms of overall frequency, there was an excess of mutations with a predicted deleterious effect on receptor function in the schizophrenia group. Bioinformatics predictions often produce divergent results [43], and none of our case-specific variants was unanimously predicted to be deleterious. Our *in vitro* analyses of the mGluR1receptor function of the case-specific mutants aimed to resolve these inconsistencies and possibly clarify genotype-phenotype correlations. P729T was also included, as a polymorphism with strong deleteriousness predictions yet no association with disease (allele frequencies 0.014 in cases and 0.019 in controls). Experimental verification of the predictions for the control-specific nsSNPs was considered to be outside the scope of this study. Moreover, most were classified as benign with very good agreement between the different bioinformatics programs.

In order to obtain functional data, we performed *in vitro* assays where we expressed wild-type and mutant mGluR1 in COS-7 cells. We analysed the major mGluR1 inositol phosphate signalling pathway after stimulation of the receptor with a glutamate agonist. To avoid receptor desensitization as a consequence of continuous stimulation by background glutamate, the cells were co-transfected with the glutamate EAAC1 transporter. The analysis revealed reduction in inositol phosphate production after receptor stimulation for 4 of the 6 case-specific mutations. The observed decrease was not due to reduced expression of the mutant proteins on the cell membrane and thus can be classified as genuine reduction of signalling function caused by the F122L, A683E, P970L and P1015A mutations. Although there are obvious differences between a COS-7 cell in an *in vitro* cell culture system and a neuron in a living human brain, the observed reduction in receptor signalling capacity indicates a potential mechanism by which these mutations could contribute to reduced mGluR1 activity *in vivo*.

F122L is located in ‘helix B’ of the mGluR1 ligand binding domain 1 (LB1), according to the published crystal structure of this region [1]. We therefore speculate that this mutation influences ligand binding or, alternatively, interferes with the activation of the receptor upon ligand binding, as suggested for an experimentally induced mutation at residue 120, I120A [44]. The N-terminal region of intracellular loop 2 contains the well-characterized ‘DRY’ motif critical for G protein-coupling in Family A GPCRs [45]. The location of A683E in this same region indicates that it is highly likely to influence the mGluR1-G protein interaction. Furthermore, the introduction of a negatively charged amino acid by the mutation, and therefore capacity to form strong electrostatic interactions, is a substantial change at the molecular level. P970L and P1015A are both present in the intracellular C-terminal tail, and as such may also play a role in G protein coupling, probably indirectly via a conformational change in protein structure. Indeed, evidence for Gβγ interaction with the C-terminal tail of mGluR7 has been published [46].

Mutation P1014S appears to affect the capacity of the receptor to be optimally expressed at the cell surface. Its intracellular localization and potential mechanisms of impaired trafficking are still to be investigated. Notably, this amino acid change did not completely abolish receptor expression, indicating partial retardation of receptor trafficking and/or a relatively subtle effect on receptor folding that may in turn contribute to receptor instability at the membrane. In the utilized *in vitro* system, the reduced expression appears not to be substantial enough to exceed receptor reserve at the plasma membrane and is therefore not reflected in reduced signalling output from the receptor population under examination. This is not an unusual phenomenon for GPCRs that often only require a fraction of receptors to be occupied in order to achieve maximal signalling efficacy, the level of which will change for different agonists and for different cellular backgrounds [47]. However, this result does point to the possibility that, by influencing receptor folding and/or trafficking, this amino acid change may contribute to suboptimal receptor function. The result for Y632H is inconclusive, due to high inter-experimental variability. Whether this reflects subtle changes in the cellular milieu affecting the expression of this mutant is unclear, but hints at further complexity of the receptor system. No significant functional effect was observed for the P729T polymorphism.

The interpretation of these *in vitro* data should also take into account the fact that the mutations occurred in the heterozygous state. Their effect *in vivo* could be mitigated by the availability of normal copies of the protein which may be sufficient for receptor activity, as discussed above in the context of reduced membrane expression of the P1014S mutant. On the other hand, the mutants can interfere with the function of the wild-type protein present in the same cells, by exerting a dominant negative effect on dimerization and/or trafficking of the receptor, reviewed in [48].

Unlike the recently reported sequencing data in sporadic schizophrenia [40]–[42], none of the changes detected in this study had arisen *de novo*. Our primary analysis was not biased towards familial cases, yet all 5 *GRM1* mutations that could be checked for segregation were shown to be inherited, and four of these occurred in families affected by a range of neuropsychiatric conditions not limited to psychotic illness. A commonality of individual genes and pathways, conferring susceptibility to different neurodevelopmental disorders, has emerged from recent studies of schizophrenia, autism spectrum disorders, intellectual deficit and epilepsy [42], [49]–[55]. In the case of mGluR1, in vitro experiments and studies of animal models point to possible involvement in anxiety, depression, epilepsy, and drug addiction and alcoholism, reviewed in [56]–[58]. Our findings support such a role.

## Supporting Information

Table S1 **Primers used in the sequencing of** ***GRM1*** **.** Genomic coordinates of primers used in PCR and sequencing given in accordance with Human Genome Assembly Feb 2009 (NCBI37/hg19).
(DOC)

Table S2 **Coding** ***GRM1*** **sequence variants identified in schizophrenia and control samples.** Abbreviations: AA, amino acid; LBD, ligand-binding domain; CRD, cysteine-rich domain; TM, Transmembrane domain; ICL, intracellular loop; ECL, extracellular loop; Cter, C terminal tail; PRD, proline-rich domain in C terminal tail; ^#^Coding exons. ^&^Protein domain nomenclature is according to Prosite (http://prosite.expasy.org/) and Pfam (http://pfam.sanger.ac.uk/). *Frequencies in Europeans have been obtained from the HapMap CEU sample, and the pilot_1_CEU_low_coverage_panel of the 1000 genomes project.
(DOC)
