# Supplementary material for: Deleterious GRM1 Mutations in Schizophrenia
Source: PLoS One. 2012 Mar 20;7(3):e32849. doi: 10.1371/journal.pone.0032849 (PMC3308973; doi:10.1371/journal.pone.0032849)
Supplement: Table S1 — Primers used in the sequencing of GRM1 . Genomic coordinates of primers used in PCR and sequencing given in accordance with Human Genome Assembly Feb 2009 (NCBI37/hg19). (DOC) [file pone.0032849.s001.doc]

**Table S1. Primers used in the sequencing of *GRM1*.**

| **Fragment** | **Primer (5’->3’)** | **Coordinates chr6*** | **Size of PCR**  **fragment (bp)** |
| --- | --- | --- | --- |
| Exon 1 | F-GACGACCATTGTTGGCG | 146350521-146350538 |  |
|  | R-GAAGGGTAAACTCCGTTCCC | 146351456-146351475 | 954 |
| Exon 2 | F-ATCTTCCGTCAGTCTCAGCC | 146480340-146480359 |  |
|  | R-TCCCACTTTGGAGTTAGCAAG | 146480838-146480858 | 518 |
| Exon 3 | F-TTGATGGGTAGTAATGCTGGC | 146625615-146625635 |  |
|  | R-AGATGGCAATTTTGTCTTTGG | 146626083-146626103 | 488 |
| Exon 4 | F-TTCTGCCAGTGTCATTGCTC | 146673264-146673283 |  |
|  | R-TTTCATCTGATATTATCCCTGGAC | 146673762-146673785 | 521 |
| Exon 5 | F-TTGGCAGTAGCTGATTTAAGATG | 146678536-146678558 |  |
|  | R-TAAGCCTGGCACACCCTTTC | 146678931-146678950 | 414 |
| Exon 6 | F-CAATTCCATCCAAACTCTAAGG | 146707837-146707858 |  |
|  | R-TTATGTATACTCCTTGACATGAGGAC | 146708275-146708300 | 463 |
| Exon 7.1 | F-GGGGACATAATTGTCCAGG | 146719715-146719733 |  |
|  | R-AGCCAGATGATACAGGTGGTG | 146720551-146720571 | 856 |
| Exon 7.2 | F-CTACCTCCAGCGCCTCTTG | 146720146-146720164 |  |
|  | R-GGGGAAATGGAAGAGACAAC | 146720946-146720965 | 819 |
| Exon 8.1 | F-AGGCCTACAGCAGAGACAGC | 146754888-146754907 |  |
|  | R-CTCCTGGAGGAGCTTAAACC | 146755652-146755671 | 783 |
| Exon 8.2 | F-TTCCATGGTGGTGCACAG | 146755269-146755287 |  |
|  | R-CAGCAGCGGTCTCCTGTC | 146756036-146756055 | 791 |

**^*^** Genomic coordinates given in accordance with Human Genome Assembly Feb 2009 (NCBI37/hg19).
