# Supplementary material for: Deleterious GRM1 Mutations in Schizophrenia
Source: PLoS One. 2012 Mar 20;7(3):e32849. doi: 10.1371/journal.pone.0032849 (PMC3308973; doi:10.1371/journal.pone.0032849)
Supplement: Table S2 — Coding GRM1 sequence variants identified in schizophrenia and control samples. Abbreviations: AA, amino acid; LBD, ligand-binding domain; CRD, cysteine-rich domain; TM, Transmembrane domain; ICL, intracellular loop; ECL, extracellular loop; Cter, C terminal tail; PRD, proline-rich domain in C terminal tail; #Coding exons. &Protein domain nomenclature is according to Prosite (http://prosite.expasy.org/) and Pfam (http://pfam.sanger.ac.uk/). *Frequencies in Europeans have been obtained from the HapMap CEU sample, and the pilot_1_CEU_low_coverage_panel of the 1000 genomes project. (DOC) [file pone.0032849.s002.doc]

**Table S2**

Coding *GRM1* sequence variants identified in schizophrenia and control samples

| Chromosome 6 NCBI36-Mar 2006 | Nucleotide substitution | Exon^#^ | AA change | Protein domain^&^ | Allele freq in cases | Allele freq in controls | dbSNP 134 rs# | Frequency in Europeans* |
| --- | --- | --- | --- | --- | --- | --- | --- | --- |
| **Schizophrenia cases only (N=443)** | | | | | | | | |
| Non-synonymous | | | | | | | | |
| 146392712 | c.366C>A | 1 | F122L | LBD | 0.11% |  |  |  |
| 146761762 | c.1894T>C | 7 | Y632H | TM2 | 0.11% |  |  |  |
| 146761916 | c.2048C>A | 7 | A683E | ICL2 | 0.11% |  |  |  |
| 146796949 | c.2909C>T | 8 | P970L | PRD | 0.11% |  |  |  |
| 146797080 | c.3040C>T | 8 | P1014S | PRD | 0.11% |  |  |  |
| 146797083 | c.3043C>G | 8 | P1015A | PRD | 0.11% |  |  |  |
| Synonymous | | | | | | | | |
| 146392538 | c.192G>A | 1 | E64E | LBD | 0.11% |  |  |  |
| 146720445 | c.1524T>C | 5 | D508D | LBD | 0.11% |  |  |  |
| 146796722 | c.2682A>G | 8 | S894S | Cter | 0.11% |  |  |  |
| **Controls only (N=589)** | | | | | | | | |
| Non-synonymous | | | | | | | | |
| 146392734 | c.388A>G | 1 | I130V | LBD |  | 0.08% |  |  |
| 146522303 | c.827A>C | 2 | K276T | LBD |  | 0.08% |  |  |
| 146715228 | c.1336G>A | 4 | D446N | LBD |  | 0.08% |  |  |
| 146749759 | c.1643C>T | 6 | T548M | CRD |  | 0.08% |  |  |
| 146797246 | c.3206C>T | 8 | P1069L | PRD |  | 0.08% | rs79336287 |  |
| 146797254 | c3214C>G | 8 | P1072A | PRD |  | 0.08% | rs146753539 |  |
| Synonymous | | | | | | | | |
| 146392610 | c.264G>A | 1 | K88K | LBD |  | 0.08% | rs145861054 |  |
| 146392790 | c.444C>G | 1 | P148P | LBD |  | 0.08% | rs138794480 |  |
| 146522307 | c.831C>T | 2 | L277L | LBD |  | 0.08% |  |  |
| 146715230 | c.1338C>T | 4 | D446D | LBD |  | 0.08% | rs142264383 |  |
| 146761848 | c.1980G>A | 7 | Q660Q | ECL1 |  | 0.08% | rs138840977 |  |
| 146761875 | c.2007G>T | 7 | A669A | TM3 |  | 0.08% | rs148926588 |  |
| 146761911 | c.2043T>C | 7 | R681R | TM3 |  | 0.08% | rs143682838 |  |
| 146762208 | c.2340C>T | 7 | N780N | ICL3 |  | 0.08% | rs41285861 |  |
| 146762232 | c.2364G>C | 7 | A788A | TM6 |  | 0.08% | rs114187147 |  |
| 146762277 | c.2409C>T | 7 | P803P | TM6 |  | 0.08% |  |  |
| 146796899 | c.2859C>T | 8 | T953T | Cter |  | 0.09% | rs116471335 |  |
| 146796923 | c.2883G>A | 8 | E961E | Cter |  | 0.08% |  |  |
| 146797007 | c.2967G>T | 8 | P989P | PRD |  | 0.08% |  |  |
| 146797397 | c.3357G>C | 8 | T1119T | Cter |  | 0.17% | rs1047008 |  |
| 146797466 | c.3426G>T | 8 | S1142S | Cter |  | 0.08% | rs147521426 |  |
| **Cases & controls (N total = 1032)** | | | | | | | | |
| Non-synonymous | | | | | | | | |
| 146762053 | c.2185C>A | 7 | P729T | ECL2 | 1.35% | 1.87% | rs41305288 | 1.7% |
| 146762519 | c.2651G>A | 7 | G884E | Cter | 0.79% | 1.52% | rs362936 | 3.3% |
| 146796825 | c.2785G>A | 8 | V929I | Cter | 2.14% | 1.78% | rs2941 | 3.7% |
| 146797017 | c.2977T>C | 8 | S993P | PRD | 52% | 54% | rs6923492 | 56% |
| Synonymous | | | | | | | | |
| 146392970 | c.624C>T | 1 | D208D | LBD | 0.45% | 0.08% | rs112670841 |  |
| 146796833 | c.2793G>A | 8 | K931K | Cter | 51% | 51% | rs2942 | 54% |
| 146796962 | c.2922T>C | 8 | P974P | PRD | 0.22% | 0.08% | rs142409803 |  |
| 146797208 | c.3168T>G | 8 | G1056G | PRD | 56% | 58% | rs6923864 | 57% |
| 146797253 | c.3213T>G | 8 | P1071P | PRD | 37% | 39% | rs1047006 | 45% |
| 146797259 | c.3219G>A | 8 | Q1073Q | Cter | 0.11% | 0.08% | rs362826 |  |
| 146797535 | c.3495C>A | 8 | P1165P | Cter | 50% | 55% | rs9373491 | 54% |
